# Ethnic variation in validity of the estimated obesity prevalence using self-reported weight and height measurements

**DOI:** 10.1186/1471-2458-11-408

**Published:** 2011-05-30

**Authors:** Henriëtte Dijkshoorn, Joanne K Ujcic-Voortman, Lucie Viet, Arnoud P Verhoeff, Daan G Uitenbroek

**Affiliations:** 1 Public Health Service of Amsterdam, Department of Epidemiology, Documentation and Health Promotion, P.O. Box 2200, 1000 CE Amsterdam, the Netherlands; 2 Centre for Prevention and Health Services Research, National Institute for Public Health and the Environment, P.O. Box 1, 3720 BA Bilthoven, the Netherlands; 3 University of Amsterdam, Faculty of Social and Behavioural Sciences, Amsterdam, the Netherlands

## Abstract

**Background:**

We examined ethnic differences between levels of body mass index (BMI) based on self-reported and measured body height and weight and the validity of self-reports used to estimate the prevalence of obesity (BMI≥30 kg/m^2^) in Turkish, Moroccan, and Dutch people in the Netherlands. Furthermore, we investigated whether BMI levels and the prevalence of obesity in Turkish and Moroccan people with incomplete self-reports (missing height or weight) differ from those with complete self-reports.

**Methods:**

Data on self-reported and measured height and weight were collected in a population-based survey among 441 Dutch, 414 Turks and 344 Moroccans aged 18 to 69 years in Amsterdam, the Netherlands in 2004. BMI and obesity were calculated from self-reported and measured height and weight.

**Results:**

The difference between measured and estimated BMI was larger in Turkish and Moroccan women than in Dutch women, which was explained by the higher BMI of the Turkish and Moroccan women. In men we found no ethnic differences between measured and estimated BMI. Sensitivity to detect obesity was low and specificity was high. In participants with available self-reported and measured height and weight, self-reports produced a similar underestimation of the obesity prevalence in all ethnic groups. However, many obese Turkish and Moroccan women had incomplete self-reports, missing height or weight, resulting in an additional underestimation of the prevalence of obesity. Among men (all ethnicities) and Dutch women, the availability of height or weight by self-report did not differ between obese and non obese participants.

**Conclusions:**

BMI based on self-reports is underestimated more by Turkish and Moroccan women than Dutch women, which is explained by the higher BMI of Turkish and Moroccan women. Further, in women, ethnic differences in the estimation of obesity prevalence based on self-reports do exist and are due to incomplete self-reports in obese Turkish and Moroccan women. In men, ethnicity is not associated with discrepancies between levels of BMI and obesity prevalence based on measurements and self-reports. Hence, our results indicate that using measurements to accurately determine levels of BMI and obesity prevalence in public health research seems even more important in Turkish and Moroccan migrant women than in other populations.

## Background

Obesity is a major problem for public health. It is associated with many chronic diseases, such as cardiovascular diseases and diabetes mellitus [1]. One group of people at high risk of obesity are non-western migrants in western societies. Among these groups, the prevalence of excessive weight is often higher than among host populations [2,3]. In Europe, migrants from Turkey and Morocco are among the largest ethnic minority groups. Turkish migrants mainly live in Germany and the Netherlands and Moroccan migrants in France, Belgium and the Netherlands [4]. In Amsterdam (the Netherlands), Turkish and Moroccan migrants represent 5% and 9% respectively of the total population [5]. Earlier studies have reported that Turkish and Moroccan migrant groups, in particular women, are at high risk of overweight and obesity [6-10]. Therefore, continued monitoring of the prevalence of obesity in these population groups is of great importance. However, although Turks and Moroccans constitute a substantial part of migrant populations in many European countries, they are often poorly represented in epidemiological studies [11]. Objective data based on physical examinations to back up self-reports are scarce [12] and available data are limited to a selected subpopulation, for example, the Turks, mothers of young children or young adults [7,9,13].

Many population-based surveys use self-reported body weight and height to determine the prevalence of obesity, mainly because of financial and logistical limitations. However, since people tend to under-report their body weight and to over-report their body height, this most likely results in an underestimation of obesity prevalence [14-18]. Several studies have suggested that the validity of self-reported body weight and height to estimate the prevalence of obesity differs across cultures [19-23]. The validity of the use of self-reports to estimate obesity prevalence rates among Turkish and Moroccan adult migrants has not previously been reported, whereas there are further concerns about its validity, because the percentage of missing self-reports among Turkish and Moroccan migrants appears to be higher compared with the Dutch population, in particular among low educated and elderly migrant women [8]. This might affect the estimated obesity prevalence considering the association of overweight with education and age [12].

This study aims to examine ethnic differences between levels of BMI based on self-reported and measured body height and weight and the validity of self-reports to estimate obesity prevalence in Turkish, Moroccan, and Dutch people in the Netherlands. Furthermore, we have investigated whether BMI levels and obesity prevalence in Turkish and Moroccan people with missing self-reported height or weight differ from those with complete self-reports. As such, our study provides new evidence for public health researchers and policymakers about ethnic variation in the validity of self-reported weight and height to estimate the obesity prevalence.

## Methods

### Study population

Data from a Dutch multiethnic population-based survey, the Amsterdam Health Monitor 2004, were examined [24]. In this health monitor both self-reported and measured data on body weight and height were collected in 2004 as part of a general health survey among the adult population (18+). The study was conducted by the Public Health Service of Amsterdam in collaboration with the National Institute for Public Health and the Environment. The study sample was drawn from the municipal register of five districts in Amsterdam. The populations from these five districts combined was representative of the total Amsterdam population. The sample was stratified by age (18-34 years; 35-44 years; 45-54 years; 55-64 years and 65 years and older) and ethnicity (Dutch, Turkish, Moroccan, and others). The individuals in the sample were invited to come to an interview and a medical examination in a local childcare center. All interviews were conducted in the language of choice of the respondent i.e. Dutch, Turkish, Moroccan-Arabic or Berber. The interviews were based on a structured questionnaire that was translated forwards and backwards from Dutch to Turkish and Arabic by certified translators.

The final response rate was 45%; Dutch 46%, Turkish 50%, and Moroccan 39%. In total 1,736 inhabitants of Amsterdam participated, of whom 518 were Dutch, 446 Turkish and 365 Moroccan. Because of the low numbers of participants aged 70 years and over (n = 93), especially among migrant groups, these people were excluded from analyses. Participants with missing data on age, measured body weight or height, pregnant women and participants with out of range data (n = 37) on body weight (< 25 kg or > 300 kg), height (< 50 cm or > 250 cm) or BMI (< 14 kg/m^2 ^or > 60 kg/m^2^) were also excluded. Consequently, the final study population consisted of 1,199 participants aged 18 to 69 years of whom 441 Dutch, 414 Turkish and 344 Moroccan.

Informed consent was obtained from participants during the health examination. Participants received written information including an explanation that the measurement of weight and height was part of the physical examination. The study protocol was approved by the Medical Ethical Committee of the Academic Medical Centre, University of Amsterdam.

### Measurements

Among a wide variety of health-related items, body height (in cm) and weight (in kg) were recorded in the questionnaire. The measurements in the health examination were taken by a trained nurse and took place immediately following the interview. Participants were dressed in light indoor clothing with emptied pockets and no shoes or hats. Height was measured without shoes, with the respondent looking straight ahead, heels connecting and feet at an angle of 45° with a wall mounted stadiometer to the nearest 0.5 cm. Weight was measured to the nearest 0.5 kg on calibrated analogue scales. To adjust for the weight of clothing, one kilogram was subtracted from the body weight. BMI was computed as weight divided by squared height in kg/m^2^. Obesity was defined as BMI ≥ 30.0 kg/m^2^, according to WHO-criteria [1]. Both the true obesity prevalence based on measured body weight and height and the estimated obesity prevalence based on self-reported weight and height were calculated. Data on socioeconomic and demographic background concerned gender, age, highest level of education (primary school or less vs. secondary education or higher), income level (below standard vs. higher; standard is net household income €1,350.00 per month), and ethnicity (Dutch, Turkish, and Moroccan). Dutch was defined as people with both parents born in the Netherlands. Turkish and Moroccan ethnicity comprised people themselves born in Turkey or Morocco or at least one of their parents. All countries of birth referred to the self-reported country of birth. To guarantee the correctness of the data, all entered data were checked immediately after data entry [24].

### Statistical analysis

For the total study population, descriptive statistics were computed, including mean measured and self-reported body weight, height and BMI, true and estimated obesity prevalence and number of missing items by gender and ethnic group. A Chi-squared test was performed to study differences between the true obesity prevalence of participants with and without available self-reports. In further analyses, participants with incomplete self-reports were excluded. A paired *t*-test was used to examine differences between self-reported and measured body weight, height and BMI, after assessing normality. Because of unequal variances, the Kruskall Wallis Test was used to examine ethnic differences. Ethnic differences in true and estimated obesity prevalence were studied with a Chi-squared test. Sensitivity and specificity for determining obesity from self-reported data were calculated. The association of Turkish or Moroccan ethnicity (ref: Dutch) with the difference between self-reported and measured BMI as dependent variable (model 1) was analyzed with linear regression analyses separately for men and women. Subsequently, age was added in model 2, measured BMI in model 3 and educational level (ref: secondary education or higher) in model 4. All statistical analyses were performed with SPSS Windows 17.0.

## Results

### Characteristics of the study population

The crude characteristics of all 1,199 participants are summarized in table 1. Moroccan men were older than their Dutch counterparts and Turkish and Moroccan women younger. Educational and income level were relatively low in the Turkish and Moroccan compared to the Dutch participants. Table 1 also shows mean self-reported and measured body height, weight, BMI, and obesity prevalence rates based on self-reported and measured data. Turkish and Moroccan women had a lower measured and self-reported height than Dutch women in our sample, whereas measured and self-reported body weight and BMI were higher. In men, no ethnic differences in measured and self-reported body weight were found. However, mean measured and self-reported body height was lower and BMI was higher in Turkish and Moroccan men than in Dutch men in our sample. In both men and women, true and estimated obesity prevalence rates were higher among Turks and Moroccans than among Dutch. Furthermore, the relative and absolute number of incomplete self-reported data are displayed in table 1. BMI could not be estimated from self-reports in 72 males (13%) and 136 females (21%), because of missing weight or height by self-report. Self-reported body height was missing more often than self-reported body weight. The percentage of missing data was highest among Moroccan women, Turkish women and Moroccan men.

**Table 1:** Characteristics of the study population by sex and ethnic group

	Dutch	Turkish	Moroccan	
***Men***	(n = 183)	(n = 197)	(n = 184)	p
**Demography**				
age (y ± sd)	47.3 (12.3)	48.3 (12.6)	51.4 (12.6)	0.003
≤ primary education	13.3%	56.6%	63.3%	<0.001
low income^a^	24.9%	72.6%	82.7%	<0.001
**Anthropometry**				
*height*				
measured (cm ± sd)	179.6 (7.0)	168.6 (6.5)	171.6 (6.6)	<0.001
self-reported (cm ± sd)	180.5 (7.2)	170.4 (6.6)	172.6 (6.8)	<0.001
missing self-reports (n)	1.6% (3)	8.6% (17)	16.8% (31)	<0.001
*weight*				
measured (kg ± sd)	81.0 (13.0)	78.5 (12.3)	79.0 (12.3)	0.136
self-reported (kg ± sd)	81.0 (12.3)	79.4 (12.1)	79.0 (12.1)	0.240
missing self-reports (n)	3.3% (6)	6.1% (12)	10.9% (20)	0.013
*BMI*				
measured (kg/m^2^± sd)	25.1 (4.0)	27.6 (4.3)	26.8 (3.8)	<0.001
self-reported (kg/m^2^± sd)	24.9 (3.7)	27.3 (4.0)	26.7 (3.6)	<0.001
missing self-reports (n)	4.4% (8)	11.7% (23)	22.3% (41)	<0.001
*Obesity*				
true (measured)	12.6%	26.4%	20.7%	0.003
estimated (self-reported)	9.1%	23.0%	16.8%	0.002

***Women***	(n = 258)	(n = 217)	(n = 160)	
**Demography**				
age (y ± sd)	48.2 (13.0)	43.0 (13.0)	43.5 (13.9)	<0.001
≤ primary education	19.1%	64.5%	63.9%	<0.001
income below standard^a^	34.8%	76.7%	76.2%	<0.001
**Anthropometry**				
*Height*				
measured (cm ± sd)	166.8 (7.0)	156.9 (5.4)	159.5 (6.4)	<0.001
self-reported (cm ± sd)	167.6 (6.9)	159.7 (5.9)	163.0 (6.5)	<0.001
missing self-reports (n)	1.2% (3)	19.8% (43)	35.0% (56)	<0.001
*Weight*				
measured (kg ± sd)	71.8 (14.4)	74.7 (14.6)	74.8 (16.1)	0.021
self-reported (kg ± sd)	71.5 (13.6)	75.0 (14.6)	74.3 (14.9)	0.010
missing self-reports (n)	6.6% (17)	9.7% (21)	19.4% (31)	<0.001
*BMI*				
measured (kg/m^2^± sd)	25.8 (5.0)	30.4 (6.0)	29.5 (6.3)	<0.001
self-reported (kg/m^2^± sd)	25.6 (4.8)	28.9 (5.8)	28.2 (6.1)	<0.001
missing self-reports (n)	7.4% (19)	23.5% (51)	41.3% (66)	<0.001
*Obesity*				
true (measured)	19.8%	49.8%	47.5%	<0.001
estimated (self-reported)	16.3%	37.3%	35.1%	<0.001

^a^net household income below € 1,350.00 per month.

Table 2 demonstrates that the obesity prevalence was higher among Turkish (73%) and Moroccan (58%) women with incomplete self-reports than among Turkish (43%) and Moroccan (40%) women with complete self-reports (Turkish: p < 0.001; Moroccan: p < 0.05; Chi-squared test). There were no statistically significant differences in the obesity prevalence of men and Dutch women with and without completed self-reports. Low educated Moroccan and Turkish women had incomplete self-reports more often than Moroccan and Turkish women with a minimum educational level of low vocational training (p < 0.001; Chi-squared test, not in table). Incompleteness of self-reported BMI was not associated with measured BMI (OR = 1.01; 95%-CI: 0.98-1.05, not in table).

**Table 2:** Obesity prevalence (%) in participants with complete and incomplete self-reports^a ^by ethnicity and gender

	Men			Women		
	Dutch	Turkish	Moroccan	Dutch	Turkish	Moroccan
	n = 183	n = 197	n = 184	n = 258	n = 217	n = 160
Participants with complete self-reports	12%	26%	21%	19%	43%	40%
Participants with incomplete self-reports	25%	26%	20%	32%	73%	58%
Chi^2^-test	0.278	0.971	0.838	0.179	<0.001	0.032

^a ^Self-reported height and/or weight.

### Use of self-reports to determine obesity prevalence

In table 3 the differences between self-reported and measured weight, height and BMI are presented. We analyzed data from 991 participants with available self-reported and measured body weight and height. In all subgroups, measured body height was lower than self-reported body height, although in Moroccan men the difference was not statistically significant. The differences between self-reported and measured body height varied between the three ethnic groups, both in men and women. The largest differences were found in Turkish (-2.5 cm) and Moroccan (-2.2 cm) women. There were no statistically significant ethnic differences between measured and self-reported body weight. Obese participants under-reported their body weight by 0.6 kg and their BMI by 1.1 kg/m^2^, with no significant differences between the ethnic groups (not in table). In most subgroups true BMI was higher than estimated BMI. The differences between true and estimated BMI were larger among Turkish and Moroccan women than among Dutch women. No ethnic differences between true and estimated BMI were found among men.

**Table 3:** Difference between measured and reported weight, height, BMI and obesity prevalence by ethnicity and sex^a^

	Dutch	Turkish	Moroccan	
***Men***	(n = 175)	(n = 174)	(n = 143)	p**^b^**
height difference (cm ± sd)	-0.8 (2.3)***	-1.8 (3.4)***	-0.6 (3.7)	<0.001
weight difference (kg ± sd)	-0.1 (3.5)	-0.5 (3.3)	0.0 (3.4)	0.333
BMI difference (kg/m^2 ^± sd)	0.2 (1.4)	0.4 (1.5)***	0.2 (1.6)	0.074
obesity difference (%,95%CI)	2.9 (1.3;6.6)	3.4 (1.6;7.3)	4.2 (1.9;8.9)	0.809
underestimation^c ^(%,95%CI)	24.2 (18.5;31.1)	12.9 (8.7;18.7)	20.0 (14.3;27.3)	0.511
***Women***	(n = 239)	(n = 166)	(n = 94)	
height difference (cm ± sd)	-0.7 (1.9)***	-2.5 (3.0)***	-2.2 (3.8)***	<0.001
weight difference (kg ± sd)	0.0 (2.4)	-0.4 (2.9)	-0.2 (3.9)	0.203
BMI difference (kg/m^2 ^± sd)	0.2 (1.1)**	0.8 (1.8)***	0.7 (2.3)**	<0.001
obesity difference (%,95%CI)	2.5 (1.1;5.4)	5.5 (2.9;10.1)	5.3 (2.3;11.8)	0.262
underestimation^c ^(%,95%CI)	13.3 (9.6;18.2)	12.9 (8.6;18.9)	13.1 (7.7;21.4)	0.994

^a^analyses limited to participants with available data on measured and self-reported height and weight.

^b^p-value of Kruskall Wallis Test on significant differences between ethnic groups.

^c^underestimation of the obesity prevalence by self-reports as a percentage of the true prevalence

*p< 0.05; **p< 0.01; ***p< 0.001 for paired *t*-test on differences between measured and self reported values.

Table 3 also shows that in participants with completed self-reports, the method of self-reported body weight and height resulted in an underestimation of obesity prevalence, varying from 2.5% in Dutch women to 5.5% in Turkish women with no statistically significant differences between the three ethnic groups. Calculated as a percentage of the true obesity prevalence, the underestimation varied from 13% in Turkish men and Dutch and Moroccan women to 24% in Dutch men.

Table 4 explores the sensitivity and specificity for detecting obesity in the three ethnic groups. Sensitivity (78%; 95%-CI: 72-85%) was fairly comparable in the three ethnic groups, but lower among men (69%;95%-CI: 57-81%) than women (84%;95%-CI: 76-91%). Highest sensitivity for obesity was found in Dutch women (87%; 95%-CI: 74-99%). Specificity for obesity was high (98%;95%-CI: 96-99%) and hardly differed between the ethnic groups. The highest specificity was found in Dutch men (99%; 95%-CI: 98-101%) and women (100%; 95%-CI:100-100%).

**Table 4:** Classification of participants into obese or non-obese based on self-reported and measured height and weight^a^

Ethnicity	Sex	Estimated obesity	True obesity		Sensitivity (95%-CI)	Specificity (95%-CI)
			no	yes		
Dutch	men	no	153	6	71% (47-96%)	99% (98-101%)
	(n = 175)	yes	1	15		
	women	no	194	6	87% (74-99%)	100% (100-100%)
	(n = 239)	yes	0	39		
Turkish	men	no	120	14	70% (53-87%)	94% (88-99%)
	(n = 174)	yes	8	32		
	women	no	92	12	83% (72-94%)	97% (92-101%)
	(n = 166)	yes	3	59		
Moroccan	men	no	109	10	67% (45-88%)	96% (92-101%)
	(n = 143)	yes	4	20		
	women	no	54	7	82% (66-97%)	96% (90-103%)
	(n = 94)	yes	2	31		
All	men	no	382	30	69% (57-81%)	97% (95-99%)
		yes	13	67		
	women	no	340	25	84% (76-91%)	99% (97-100%)
		yes	5	129		
	total	no	722	55	78% (72-85%)	98% (96-99%)
		yes	18	196		

^a^analyses limited to participants with available data on both measured and self-reported weight and height.

Table 5 presents the results from linear regression analyses. These showed that among men, the difference between true and estimated BMI increased with age and was not associated with ethnicity and educational level (table 5). After including measured BMI in the model, the association with age was no longer significant. Among Turkish and Moroccan women, the difference between true and estimated BMI was larger than among Dutch women and increased with age. The addition of measured BMI to the model explained the ethnic differences and the association with age was no longer significant. Educational level had no effect on the association between ethnicity and the difference between true and estimated BMI.

**Table 5:** Multivariate linear regression analysis of the association between ethnicity and difference between measured and estimated BMI

	Men	Women
	B, 95% CI	B, 95% CI
*Model 1*		
Turkish	0.23 (-0.09;0.55)	0.56 (0.25;0.88)**
Moroccan	-0.03 (-0.36; 0.31)	0.47 (0.08;0.85)*
Dutch	ref	ref
*Model 2: adjusted for age*		
Turkish	0.21 (-0.10;0.53)	0.67 (0.34;0.99)***
Moroccan	-0.08 (-0.42;0.26)	0.60 (0.20;0.99)**
Dutch	ref	ref
Age	0.01 (0.00;0.02)*	0.02 (0.00;0.03)**
*Model 3: adjusted for age, measured BMI*		
Turkish	-0.08 (-0.40;0.23)	0.25 (-0.09;0.58)
Moroccan	-0.25 (-0.58;0.07)	0.22 (-0.17;0.61)
Dutch	ref	ref
age	0.00 (-0.01;0.01)	0.0 (-0.01;0.01)
measured BMI	0.12 (0.08;0.15)***	0.09 (0.06;0.11)***
*Model 4: adjusted for age, measured BMI and educational level*		
Turkish	-0.16 (-0.50;0.19)	0.16 (-0.21;0.52)
Moroccan	-0.33 (-0.68;0.03)	0.20 (-0.22;0.61)
Dutch	ref	ref
age	0.0 (-0.01;0.01)	0.00 (-0.01;0.01)
measured BMI	0.12 (0.09;0.16)***	0.08 (0.06;0.11)***
educational level	0.12 (-0.18;0.43)	0.22 (-0.10;0.55)

B, 95% CI, multivariate linear regression coefficient with 95% confidence interval.

*p < 0.05, **p < 0.01, ***p < 0.001 for comparison with Dutch ethnic group, ref = reference.

## Discussion

This study investigated ethnic differences between BMI based on self-reported and measured body height and weight and the validity of self-reports for estimating the prevalence of obesity among Turks, Moroccans and Dutch in the Netherlands. It showed that among Turkish and Moroccan women, the accuracy of BMI based on self-reports was less than among Dutch women, which could be explained by the higher BMI among Turkish and Moroccan women. Among men, there was no association of ethnicity with the accuracy of BMI based on self-reports. Among women, we also found ethnic differences in the estimation of the prevalence of obesity based on self-reports, due to incomplete self-reports in obese Turkish and Moroccan women. In men, ethnicity was not associated with discrepancies between obesity prevalence based on measurements and self-reports. The sensitivity and specificity for detecting obesity from self-reports was comparable between ethnic groups.

### Strengths and limitations

When interpreting the results of our study its strengths and limitations should be taken into account. Important strengths are the inclusion of a large sample of two well-defined ethnic minority groups, Turks and Moroccans, who could be compared to Dutch people from the same study population. Further, we had the unique opportunity to compare the validity of self-reported data on body weight and height with data collected in a physical examination among these three ethnic groups.

One limitation was the fact that participants received written information on the measurements, which was required by the local Medical Ethical Committee. Therefore, we cannot exclude the possibility of self-reports being affected by the foreknowledge of the participants that they would be measured. However, we assume this influence was small, because the comprehensive health interview was completed prior to the health examination, which included a blood sample and a variety of other measurements. Moreover, the participants received written information one week prior to the health examination, whereas the oral explanation on the health examination was given after the interview.

Another potential source of bias could have resulted from the relatively low response rate and the limited number of participants in the ethnic subgroups. The response rate of the survey is comparable to that of other surveys in the Netherlands [10,25,26]. We therefore believe that these relative low response rates were not caused by a specific flaw in the study's design and are unlikely to indicate systematic bias. In addition, the number of individuals who did not receive their invitation because of incorrect residential addresses in the municipal registers is likely to be high because of the mobility of the population in Amsterdam [27]. Therefore, our actual response rate might be higher. We analyzed the possibility of selective non-response by age and socio-economic status and concluded that overall, income level as well as level of unemployment in our sample were comparable to that in the total Amsterdam population [28]. The response rates by age and ethnic groups were also analyzed and compared to the Dutch. We found comparable response rates among 18 to 69-year-old participants by ethnicity [24]. Because a non-weighted age-stratified sample was used for the analyses, the age groups were equally represented in each of the ethnic groups. Although the number of participants in each ethnic subgroup was relatively small, it was still large enough to demonstrate ethnic differences between measured and self-reported BMI-differences in women, but not large enough to show ethnic differences between true and estimated obesity prevalence. Among men, ethnic differences between true and estimated obesity prevalence were small, were not statistically significant and in our opinion had no clinical relevance. The same applied for ethnic differences between measured and self-reported BMI-differences in men.

Finally, as many comparisons were made, the findings in our study might have been caused by chance fluctuation. Post-hoc analyses were not applied as we basically tested pre-existing hypotheses and did not dredge the data for new findings. However, our findings need confirmation in a follow up study.

### Fit with previous evidence and explanations

To our knowledge, this is the first study of discrepancies in BMI and obesity prevalence among adult men and women of Turkish and Moroccan origin. Previous studies on this topic among other immigrant groups showed that the underestimation of BMI and the obesity prevalence based on self-reports varies by ethnicity and gender. In Mexican American men and women and in African American women, discrepancies between measurements and self-reports were larger than in European Americans. However, among men and women of South Asian origin (Hindustani-Surinamese) and African-Surinamese women, discrepancies were smaller than among the Dutch [20-22].

In our study, the accuracy of self-reports was smaller among Turkish and Moroccan women than among Dutch women. Furthermore, the obesity prevalence among Turkish and Moroccan women with missing height or weight when using self-reports was higher than obesity among Turkish and Moroccan women with completed self-reports. Low educated Moroccan and Turkish women had incomplete self-reports more often than Moroccan and Turkish women with a minimum educational level of low vocational training. The single use of self-reported height and weight would have resulted in an underestimation of the obesity prevalence among Turkish women by 30% and among Moroccan women by 18%. This issue was not described in the studies mentioned above [20-22]. Previous studies on the validity of self-reported weight and height among adolescents reported a high percentage of missing self-reports. As a possible explanation, the authors mentioned that the rapid growth of people during adolescence combined with the lack of recent measurements makes respondents less likely to know their current weight and height [29-33].

In our study, possible explanations for the high percentage of mis-reports and incomplete self-reports include the higher prevalence of obesity among migrant women compared with Dutch women and the tendency of obese people to under-report their weight more than non-obese people [18]. Further recall bias might play a role, related to the high illiteracy rate and low educational level in Turkish and Moroccan migrant women [34]. This is a plausible reason, as the inability to read and spell in low-income women has also been shown to be associated with a less accurate recall of their food intake [35]. Also, a lack of recent measurements might play a role. Regular measurements are important for both weight and height, because weight gain and a loss of height accompanies aging. As a result, respondents might be less likely to know their current weight and height [16]. Furthermore, reporting height and weight might be biased towards cultural ideals [20]. Because Turkish and Moroccan women over-reported weight, we believe that social desirability in answering did not play a large role in the assumption that the western ideal of a thin body size also applies to these groups.

## Conclusions

The use of self-reported weight and height leads to an underestimation of BMI and obesity prevalence, both in the Dutch population and in Turkish and Moroccan migrant groups in the Netherlands. BMI based on self-reports is more likely to be underestimated in Turkish and Moroccan women than in the Dutch although this could be explained by their higher BMI. Among Turkish and Moroccan women, there is an additional underestimation of the obesity prevalence based on self-reports, because obese Turkish and Moroccan women have incomplete self-reports more often than those who are non-obese. In men, ethnicity is not associated with discrepancies between BMI and obesity prevalence based on self-reports and measurements. In addition to the general recommendation from previous studies to use measurements instead of self-reports to validly determine BMI and obesity prevalence in public health research, our results indicate that the use of measurements seems even more important in Turkish and Moroccan migrant women.

## Competing interests

The authors declare that they have no competing interests.

## Authors' contributions

HD was responsible for statistical analyses and interpretation of data. JU participated in the study concept, design and coordination as well as participating in analyses and interpretation of data and was also involved in critical revision of the manuscript. LV participated in the study concept, design and coordination and was also involved in critical revision of the manuscript. AV participated in the study concept and design and was involved in critical revision of the manuscript. DU participated in the study concept, design and coordination, participated in analyses and interpretation of data and was also involved in critical revision of the manuscript. All authors read and approved the final manuscript.

## Pre-publication history

The pre-publication history for this paper can be accessed here:

http://www.biomedcentral.com/1471-2458/11/408/prepub
